# Heat stress elicits remodeling in the anther lipidome of peanut

**DOI:** 10.1038/s41598-020-78695-3

**Published:** 2020-12-17

**Authors:** Zolian S. Zoong Lwe, Ruth Welti, Daniel Anco, Salman Naveed, Sachin Rustgi, Sruthi Narayanan

**Affiliations:** 1 Department of Plant and Environmental Sciences, Clemson University, Clemson, SC USA; 2 Division of Biology, Kansas State University, Manhattan, KS USA; 3 Edisto Research & Education Center, Clemson University, Blackville, SC USA; 4 Pee Dee Research & Education Center, Clemson University, Florence, SC USA

**Keywords:** Heat, Plant physiology, Lipidomics

## Abstract

Understanding the changes in peanut (*Arachis hypogaea* L.) anther lipidome under heat stress (HT) will aid in understanding the mechanisms of heat tolerance. We profiled the anther lipidome of seven genotypes exposed to ambient temperature (AT) or HT during flowering. Under AT and HT, the lipidome was dominated by phosphatidylcholine (PC), phosphatidylethanolamine (PE), and triacylglycerol (TAG) species (> 50% of total lipids). Of 89 lipid analytes specified by total acyl carbons:total carbon–carbon double bonds, 36:6, 36:5, and 34:3 PC and 34:3 PE (all contain 18:3 fatty acid and decreased under HT) were the most important lipids that differentiated HT from AT. Heat stress caused decreases in unsaturation indices of membrane lipids, primarily due to decreases in highly-unsaturated lipid species that contained 18:3 fatty acids. In parallel, the expression of *Fatty Acid Desaturase 3-2* (*FAD3-2*; converts 18:2 fatty acids to 18:3) decreased under HT for the heat-tolerant genotype SPT 06-07 but not for the susceptible genotype Bailey. Our results suggested that decreasing lipid unsaturation levels by lowering 18:3 fatty-acid amount through reducing *FAD3* expression is likely an acclimation mechanism to heat stress in peanut. Thus, genotypes that are more efficient in doing so will be relatively more tolerant to HT.

## Introduction

Peanut (*Arachis hypogaea* L.) ranks among the top five most important oilseeds worldwide along with soybean (*Glycine max* L.), rapeseed (*Brassica napus* L.), sunflower (*Helianthus annuus* L.), and cotton (*Gossypium* spp. L.), and has seen more than 100% increase in global production since the 1970s^1,2^. China, India, Nigeria, Sudan, and the United States, in that order, are the top five producers of peanuts^2^. However, like many other crops, peanut production in most, if not all, regions is currently challenged by reduced yield due to heat stress. Peanut seed yield decreased by 14, 59, and 90% when day/night temperature increased from 32/22 °C to 36/26, 40/30, and 44/34 °C, respectively^3^. Climate models predict continued warming such that the global mean surface air temperature will increase at least by 1.5 °C between 2030 and 2052—an additional increase of 0.5 °C from today’s level^4^. Thus, the threat of heat stress to peanut production is expected to increase in the future. Therefore, development of heat-tolerant peanut varieties is essential to maintain peanut production and profitability.

Daily average temperatures over 30 °C significantly retard vegetative and reproductive growth and pod yield in peanut^5,6^. It has been reported that a daytime temperature of 38 °C caused the greatest yield reduction when stress occurred from 6 days before to 15 days after flowering^7^. This reduction was primarily due to fewer flowers producing pegs (a peg is a stalk-like structure formed at the base of the ovary, which enters the soil and forms a pod at the tip). The same authors also found that reduced pollen production and viability under high temperatures (≥ 34/28 °C, day/night) during this most sensitive stage, around the time of flowering, caused decreased fruit-set^8^. However, the mechanism of decrease in peanut pollen performance during heat stress remains largely unclear. A better understanding of these mechanisms will help develop bio- and molecular markers for better pollen performance under heat stress.

Lipids occupy multiple roles that span metabolic, regulatory, and structural domains in plant growth and development and in responses to environmental stresses^9–11^. Lipids are the major constituents of biological membranes. Membrane fluidity and stability that underpin the structure and function of cells are determined by lipid composition and fatty acid unsaturation levels^12^. In previous research, our group identified lipid metabolic changes during heat stress responses in wheat (*Triticum aestivum* L.)^13–15^. We found that wheat pollen lipid composition is significantly altered by high temperatures, and some lipids are highly heat-responsive, in particular, the extraplastid-localized phospholipids, phosphatidylcholine (PC) and phosphatidylethanolamine (PE)^15^. Wheat plants decreased pollen lipid unsaturation levels to adapt to heat stress and underwent lipid remodeling, likely to prevent the phase transition of membranes from a bilayer to a non-bilayer phase. Similar alterations occurred in the leaves of the heat-tolerant soybean genotype DS25-1^16^. DS25-1 also had significant decreases in the expression levels of the fatty-acid desaturase (FAD) genes *FAD3A* and *FAD3B*, which convert linoleic (18:2) acid to linolenic (18:3) acid.

It has been reported that pollen development in *Brassica napus* is contingent upon lipid biosynthesis in, not just pollen, but also in the tapetum (a specialized layer of nutritive cells) of anthers^17^. Ariizumi et al.^18^ found that, in *Arabidopsis*, the disruption of a novel protein, no exine formation 1 (NEF1), considerably lowered lipid accumulation in anthers and resulted in lack of exine, pollen abortion, complete absence of pollen grains at anthesis, and male sterility. Additionally, it was demonstrated that mutants of the rice (*Oryza sativa* L.) *Tapetum Degeneration Retardation (TDR)* gene or *Defective Tapetum Cell Death 1 (DTC1)* gene exhibited abnormal lipid biosynthesis or transport in anthers, defective pollen development, and male sterility^19,20^. This suggests that deleterious metabolic changes in anther can reduce pollen performance and, consequently, result in lower yield.

The objective of this study was to evaluate the anther lipidome of peanut genotypes to identify changes in lipid traits or species composition under heat stress that may confer an acclimation response. We evaluated a total of seven peanut genotypes in two growing seasons (2018 and 2019) at high temperature (HT, heat stress; 41/27 °C for 2018 and 38/26 °C for 2019, average day/night temperatures imposed for 17 days and 18 days during flowering in 2018 and 2019, respectively) and ambient temperature (AT, control; 31/22 °C for 2018 and 28/22 °C for 2019) under field conditions. We hypothesized that the anther lipidome in peanut is altered by heat stress and that some lipid responses will be associated with heat tolerance of peanut genotypes.

## Results

### Heat stress affects physiological and yield traits

**Figure 1:**
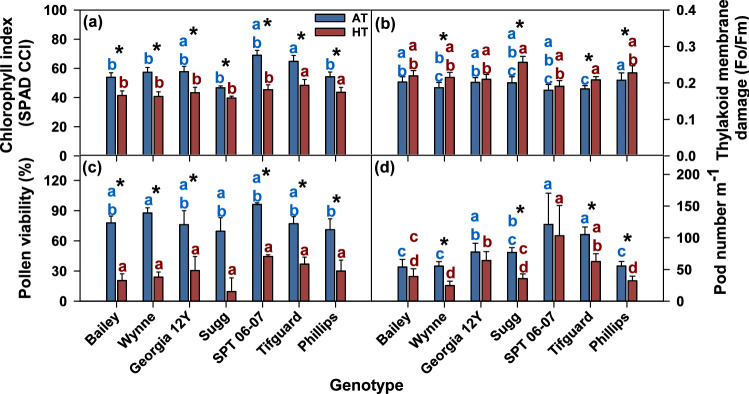
Temperature effect on chlorophyll index (**a**), thylakoid membrane damage (**b**), pollen viability (**c**), and pod number (number of pods in 1-m row length) (**d**) of peanut genotypes grown under field conditions in 2018 and 2019. Values shown are least-squares means. Error bars represent standard errors about the least-squares mean of 16 observations (2 years × 2 blocks × 4 replications) except for Sugg and SPT 06-07, which have 8 observations (1 year × 2 blocks × 4 replications). Least-squares means with different letters are significantly different according to Fisher’s least significant difference (LSD) test at α = 0.05. Blue letters compare genotypes under AT and red letters under HT. An asterisk (*) above bars indicate a significant difference at α = 0.05 between AT and HT for that genotype. AT, ambient temperature (31/22 °C for 2018 and 28/22 °C for 2019; average day/night temperatures during the 17-days and 18-days treatment periods in 2018 and 2019, respectively); HT, high temperature (41/27 °C for 2018 and 38/26 °C for 2019). Data from 2 years (2018 and 2019) were pooled together for analysis.

Heat stress (41/27 °C in 2018 and 38/26 °C in 2019) significantly affected the physiological and yield traits of all genotypes. Across the genotypes, chlorophyll index decreased by 15 to 34%, a parameter indicating thylakoid membrane damage increased by 4 to 28%, pollen viability decreased by 52 to 86%, and pod number decreased by 15 to 55% under HT (Fig. 1). Overall, Bailey, Wynne, and Sugg exhibited poor performance, whereas SPT 06-07, Georgia 12Y, and Tifguard exhibited better performance under HT in terms of the above traits. Notably, Bailey and Sugg have less than or equal to 20% viable pollen under HT, whereas Georgia 12Y, SPT 06-07, Tifguard, and Phillips had 30, 45, 37, and 30% viable pollen, respectively. Wynne was intermediate in terms of pollen viability (24% viable pollen) (Fig. 1).

### Anther lipids were profiled and quantified using an electrospray ionization-triple quadrupole mass spectrometry (ESI–MS/MS)

**Figure 2:**
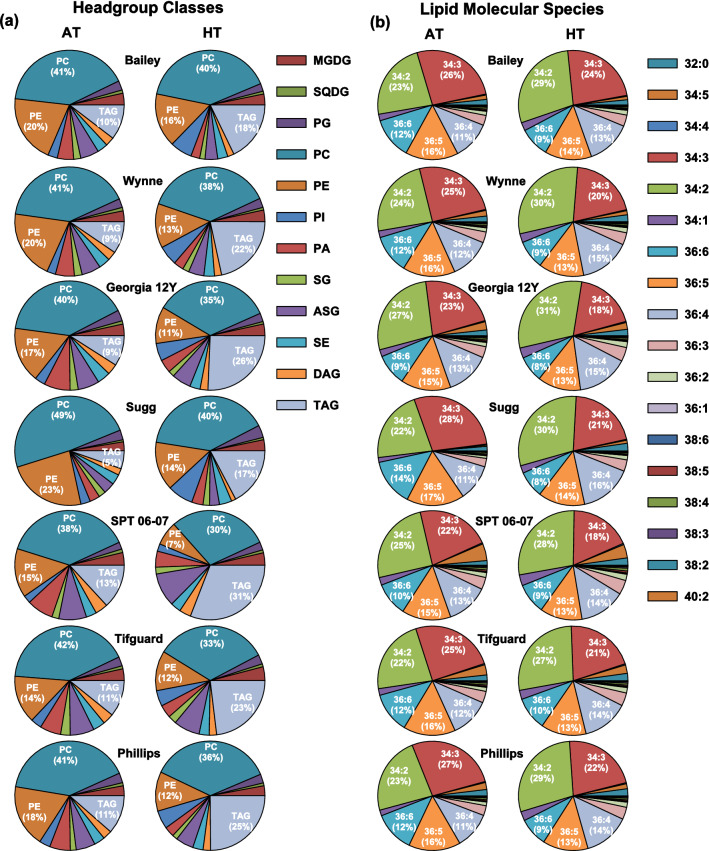
Pie charts representing composition of various lipid headgroup classes (**a**) and lipid molecular species (**b**) in peanut anthers under ambient temperature (AT, 31/22 °C for 2018 and 28/22 °C for 2019; average day/night temperatures during the 17-days and 18-days treatment periods in 2018 and 2019, respectively) and high temperature (HT, 41/27 °C for 2018 and 38/26 °C for 2019). ASG, acylated sterol glycoside; DAG, diacylglycerols; MGDG, monogalactosyldiacylglycerol; PA, phosphatidic acid; PC, phosphatidylcholine; PE, phosphatidylethanolamine; PG, phosphatidylglycerol; PI, phosphatidylinositol; SE, sterol ester; SG, sterol glycoside; SQDG, sulfoquinovosyldiacylglycerol; TAG, triacylglycerol. Lipid molecular species are identified as total acyl carbons: total double bonds. Data from 2 years (2018 and 2019) were pooled together for analysis.

Anther samples were collected during the treatment period for lipid profiling from the peanut plants on the 13th and 9th day in 2018 and 2019, respectively. The 89 lipid analytes quantified (Supplementary Table S2) belonged to the following groups: plastidic lipids [monogalactosyldiacylglycerol (MGDG), phosphatidylglycerol (PG), sulfoquinovosyldiacylglycerol (SQDG)], extraplastidic phospholipids [PC, PE, phosphatidylinositol (PI)], phosphatidic acid (PA), sterol derivatives [acylated sterol glycosides (ASG), sterol glycosides (SG), sterol esters (SE)], diacylglycerols (DAG), and triacylglycerols (TAG). Under AT, PC was the most predominant headgroup class in all genotypes (38–49%), followed by PE (14–23%) and TAG (5–13%) (Fig. 2a). Under HT, PC was still the most predominant headgroup class in all genotypes (30–40%), which was followed by TAG (17–31%) and PE (7–16%) (Fig. 2a).

When each genotype’s anther lipidome was analyzed based on the lipid molecular species composition of structural glycerolipids, five species were most abundant under AT: 34:3 (22–28%), 34:2 (22–27%), 36:6 (9–14%), 36:5 (15–17%), and 36:4 (11–13%) (Fig. 2b). The same five lipid species dominated the peanut anther lipidomes of all genotypes under HT as well, though the amounts were decreased for 34:3 (18–24%), 36:6 (8–10%), and 36:5 (13–14%) and increased for 34:2 (27–31%) and 36:4 (13–16%) under HT (Fig. 2b). Together, these five species accounted for 85 to 92% of the peanut anther lipidome under AT and 82 to 89% under HT across the genotypes. The 34:3 lipids contain highly unsaturated 18:3 fatty acid. Though the 34:3 lipids decreased in all genotypes under HT, the decrease was of smallest magnitude for Bailey.

### Heat stress alters the unsaturation levels and composition of lipid headgroup classes

**Figure 3:**
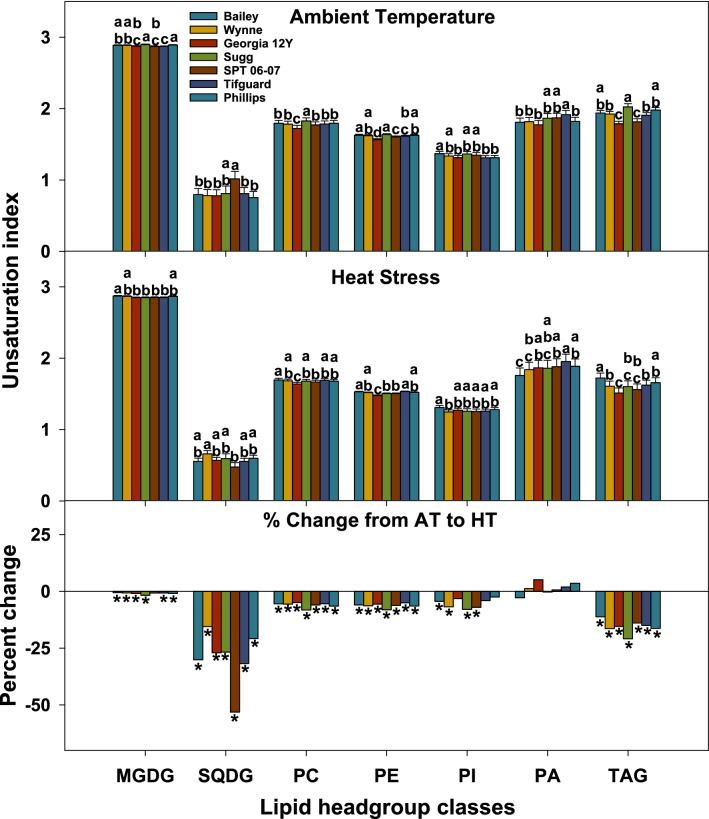
Changes in unsaturation index of various lipid classes in peanut anthers in response to heat stress. The unsaturation index for a lipid molecular species was calculated as the average number of double bonds per acyl chain, which is the number of double bonds in the lipid molecular species divided by the number of acyl chains. The unsaturation index of a lipid headgroup class was calculated as: $\frac{{\sum {\left( {{\text{unsaturation} \; \text{indices} \; \text{of} \; \text{individual} \; \text{lipid} \; \text{molecular} \; \text{species}\; \text{in} \; \text{the} \; \text{class}} \times {\text{amount} \; \text{of} \; \text{each} \; \text{species}}} \right)} }}{{\sum {{\text{amount} \; \text{of} \; \text{all}\; \text{lipid} \; \text{molecular} \; \text{species}\; \text{in} \; \text{the} \; \text{class}}} }}.$ Values shown are least squares means. Error bars represent standard errors about the least-squares mean of 16 observations (2 years × 2 blocks × 4 replications) except for Sugg and SPT 06-07, which each have 8 observations (1 year × 2 blocks × 4 replications). Least-squares means with different letters are significantly different according to Fisher’s least significant difference (LSD) test at α = 0.05. An asterisk (*) above bars indicate a significant difference at α = 0.05 between AT and HT for that genotype. AT, ambient temperature (31/22 °C for 2018 and 28/22 °C for 2019; average day/night temperatures during the 17- d and 18-d treatment periods in 2018 and 2019, respectively); HT, high temperature (41/27 °C for 2018 and 38/26 °C for 2019). MGDG, monogalactosyldiacylglycerol; PA, phosphatidic acid; PC, phosphatidylcholine; PE, phosphatidylethanolamine; PI, phosphatidylinositol; SQDG, sulfoquinovosyldiacylglycerol; TAG, triacylglycerol. Data from 2 years (2018 and 2019) were pooled together for analysis.

**Figure 4:**
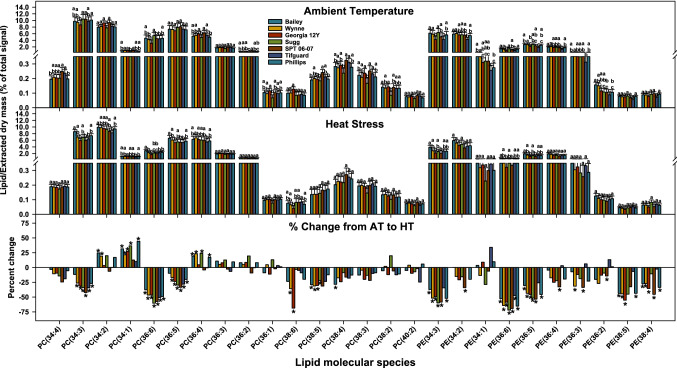
Changes in the amount of phosphatidylcholine (PC) and phosphatidylethanolamine (PE) molecular species, as percentage of total mass spectral signal, in peanut anthers in response to heat stress. Lipid species shown were those that passed both the limit of detection (LOD > 0.0005 nmol) and coefficient of variation (CoV < 0.3) cutoffs (see “Materials and methods” for more details). Values shown are least-squares means. Error bars represent standard errors about the least-squares mean of 16 observations (2 years × 2 blocks × 4 replications) except for Sugg and SPT 06-07, which have 8 observations (1 year × 2 blocks × 4 replications). Least-squares means with different letters are significantly different according to Fisher’s least significant difference (LSD) test at α = 0.05. An asterisk (*) above bars indicate a significant difference at α = 0.05 between AT and HT for that genotype. AT, ambient temperature (31/22 °C for 2018 and 28/22 °C for 2019; average day/night temperatures during the 17-days and 18-days treatment periods in 2018 and 2019, respectively); HT, high temperature (41/27 °C for 2018 and 38/26 °C for 2019). Lipid molecular species are identified as total acyl carbons: total double bonds. Breaks on the y axis indicate a change in scale. Data from 2 years (2018 and 2019) were pooled together for analysis.

Unsaturation indices of MGDG, SQDG, PC, PE, PI, and TAG decreased numerically under HT in all genotypes (most changes were statistically significant) (Fig. 3). The changes in unsaturation levels among headgroup classes are attributable to alterations in the lipid molecular species composition (Figs. 2, 4). These alterations primarily include decreases in the relative amounts of highly unsaturated lipid species (e.g., 34:3, 36:6, and 36:5) that contained 18:3 fatty acid chain (Figs. 2, 4).

**Figure 5:**
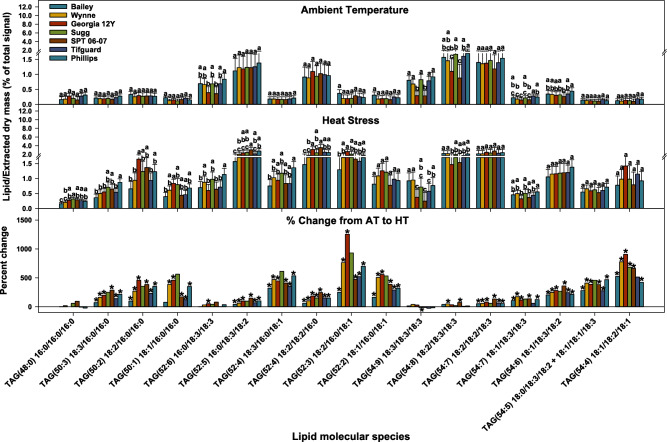
Changes in the percentage of total signal of triacylglycerol (TAG) molecular species in peanut anthers in response to heat stress. Lipid species shown were those that passed both the limit of detection (LOD > 0.0005 nmol) and coefficient of variation (CoV < 0.3) cutoffs (see “Materials and methods” for more details). Values shown are least-squares means. Error bars represent standard errors about the least-squares mean of 16 observations (2 years × 2 blocks × 4 replications) except for Sugg and SPT 06-07, which have 8 observations (1 year × 2 blocks × 4 replications). Least-squares means with different letters are significantly different according to Fisher’s least significant difference (LSD) test at α = 0.05. An asterisk (*) above bars indicate a significant difference at α = 0.05 between AT and HT for that genotype. AT, ambient temperature (31/22 °C for 2018 and 28/22 °C for 2019; average day/night temperatures during the 17-days and 18-days treatment periods in 2018 and 2019, respectively); HT, high temperature (41/27 °C for 2018 and 38/26 °C for 2019). Lipid molecular species are identified as total acyl carbons: total double bonds. Acyl-chain composition is included after each TAG lipid species but does not indicate *sn* positions. Breaks on the y axis indicate a change in scale. Data from 2 years (2018 and 2019) were pooled together for analysis.

To see how the fatty acids of the predominant lipid classes were changing in response to HT, the amount of each fatty acid in lipid molecular species that can be unambiguously assigned were summed and compared between AT and HT (Table 1). This analysis showed that overall, 18:3 was reduced to the largest extent compared to the other major fatty acids in PC and PE in all genotypes (Table 1). The overall changes for DAG acyl chains were similar to that of PC as expected considering the close metabolic relationship between DAG and PC, particularly as both are reactants in the formation of TAG (Table 1). All TAG molecular species increased significantly in response to heat treatment in percent of total lipid except TAG(16:0/16:0/16:0) and TAG(18:3/18:3/18:3) (Fig. 5 and Supplementary Table S3). This is in contrast to data from Arabidopsis leaves and seedlings, where 18:3/18:3/18:3 levels are highly increased in leaves under short-term heat stress^21–24^. SPT 06-07 had a larger percent increase under HT than Bailey for most TAG species (Fig. 5). Analysis of the fatty acids in TAG showed that all fatty acids were increasing. In contrast, 18:1 was increasing by the largest fold, whereas 18:3 had the lowest fold increase (Table 1).

**Table 1:**
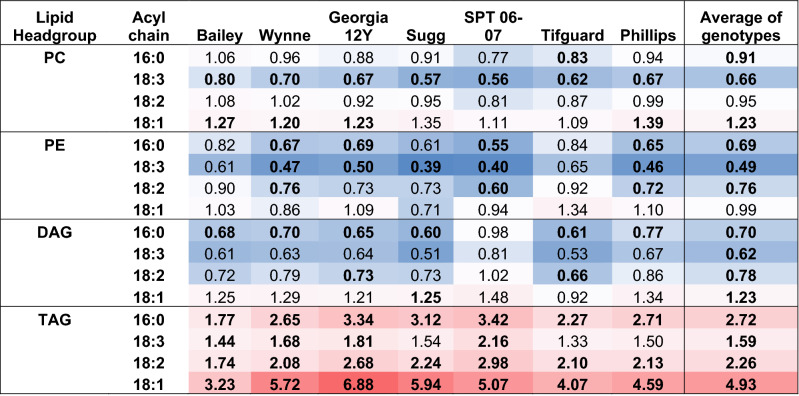
Fold change of fatty acid for HT/AT in each lipid class.

Lipid molecular species for which the fatty acyl composition is unambiguous were used in the analysis. For PC, this included 34:4 (16:1/18:3), 34:3 (16:0/18:3), 34:2 (16:0/18:1), 34:1 (16:0/18:1), 36:6 (18:3/18:3), 36:5 (18:2/18:3), and 36:1 (18:0/18:1). For PE, 34:3 (16:0/18:3), 34:2 (16:0/18:1), 34:1 (16:0/18:1), 36:6 (18:3/18:3), and 36:5 (18:2/18:3) were included. For DAG, all species were included, and for TAG, all species except TAG(54:5) were included. The fold values were calculated as the sum of (each molecular species times the number of occurrences of the fatty acid in that species) in HT/the sum of (each molecular species times the number of occurrences of the fatty acid in that species) in AT. Lipid molecular species are identified as total acyl carbons: total double bonds. Bolded numbers represent significant changes from AT to HT (α = 0.05; Supplementary Table S3). Red shades represent increase and blue shades represents decrease in fold change. AT, ambient temperature (31/22 °C for 2018 and 28/22 °C for 2019; average day/night temperatures during the 17- d and 18-d treatment periods in 2018 and 2019, respectively); HT, high temperature (41/27 °C for 2018 and 38/26 °C for 2019). DAG, diacylglycerol; PC, phosphatidylcholine; PE, phosphatidylethanolamine; TAG, triacylglycerol.

Comparing the genotypes, Bailey, unlike other genotypes, did not have a significant decrease in the amounts of both highly unsaturated PC species 34:3 and 36:5, had the smallest percent decrease in PC(36:6), and had the smallest fold decrease in 18:3-containing PC and PE (Fig. 4 and Table 1). SPT 06-07 had the largest decrease in the amounts of PC(34:3) and PC(36:5), the second largest decrease for PC(36:6), and the biggest fold decrease in 18:3-containing PC. The lipid species 34:3 and 36:5 each contain one 18:3 chain, whereas 36:6 contains two^25^.

### Partial least squares-discriminant analysis (PLS-DA) identifies the most important lipids in discriminating AT from HT using Variable Importance in Projection (VIP) Scores

**Figure 6:**
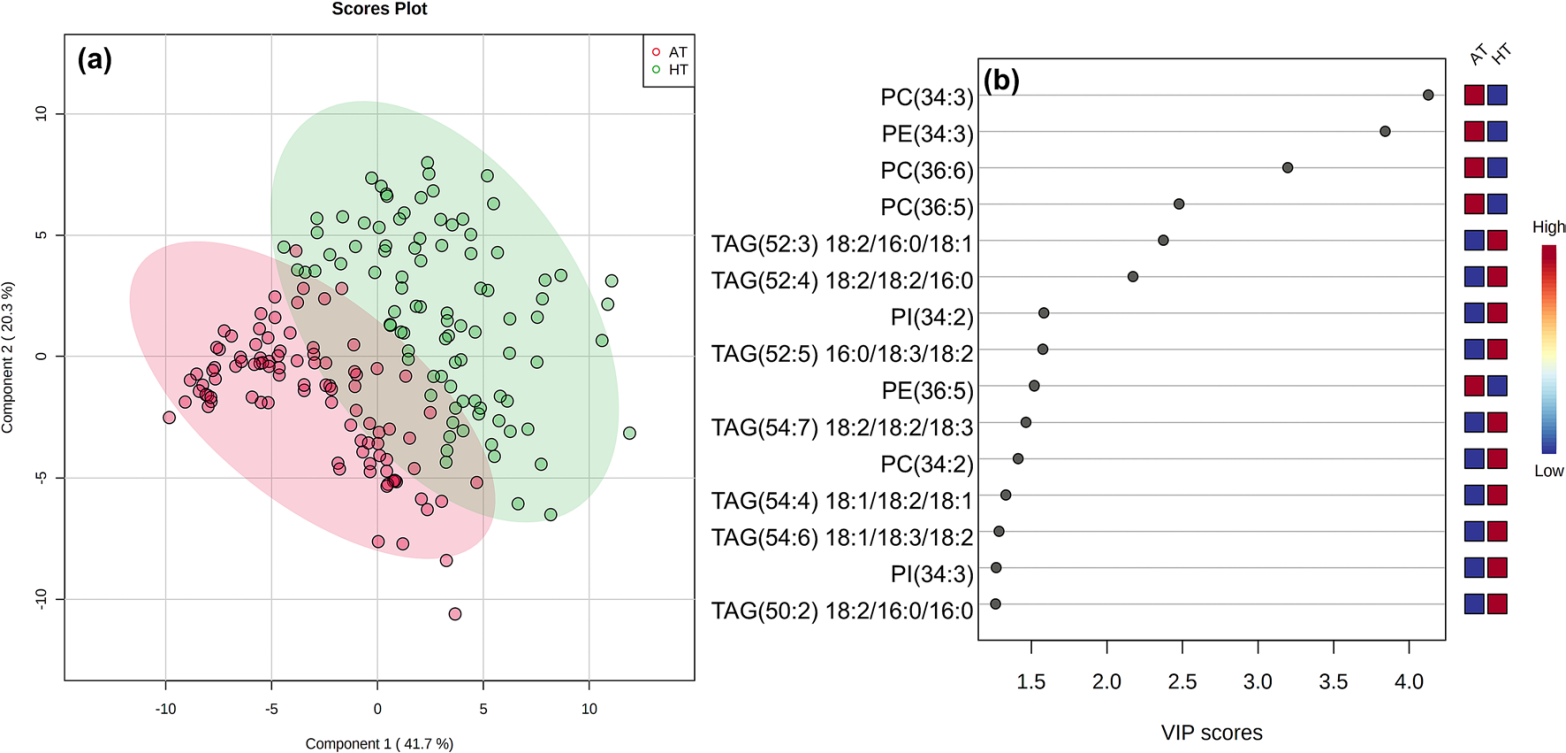
Partial least squares-discriminant analysis (PLS-DA) scores plot (**a**) demonstrating the differentiation of the two treatments: ambient temperature (AT, 31/22 °C for 2018 and 28/22 °C for 2019; average day/night temperatures during the 17-days and 18-days treatment periods in 2018 and 2019, respectively) and high temperature (HT, 41/27 °C for 2018 and 38/26 °C for 2019) and PLS-DA variable importance in projection (VIP) scores (**b**) that identified the top 15 lipid species that differentiated the treatments. A total of 89 lipid species were analyzed under the two treatments. Data from 2 years (2018 and 2019) were pooled together for analysis. PC, phosphatidylcholine; PE, phosphatidylethanolamine; PI, phosphatidylinositol; TAG, triacylglycerol. Lipid molecular species are identified as total acyl carbons: total double bonds. Acyl-chain composition is included after each TAG lipid species but does not indicate *sn* positions.

We utilized PLS-DA, a supervised learning method, to visualize the separation of AT from HT by the 89 lipid analytes (Fig. 6a) and to rank the lipid species based on their ability to differentiate AT from HT (Fig. 6b). The top 15 lipids according to their order of importance based on VIP scores were PC(34:3), PE(34:3), PC(36:6), PC(36:5), TAG(18:2/16:0/18:1) [i.e., TAG(52:3)], TAG(18:2/18:2/16:0) [i.e., TAG(52:4)], PI(34:2), TAG(16:0/18:3/18:2) [i.e., TAG(52:5)], PE(36:5), TAG(18:2/18:2/18:3) [i.e., TAG(54:7)], PC(34:2), TAG(18:1/18:2/18:1) [i.e., TAG(54:4)], TAG(18:1/18:3/18:2) [i.e., TAG(54:6)], PI(34:3), and TAG(18:2/16:0/16:0) [i.e., TAG(50:2)] (Fig. 6b). Interestingly, 6 out of the 15 lipids above were PC or PE species containing 34:3, 36:6, 36:5, and 34:2 species, and the rest were primarily TAG species.

### PC and PE molecular species undergo coordinated metabolism under HT

**Figure 7:**
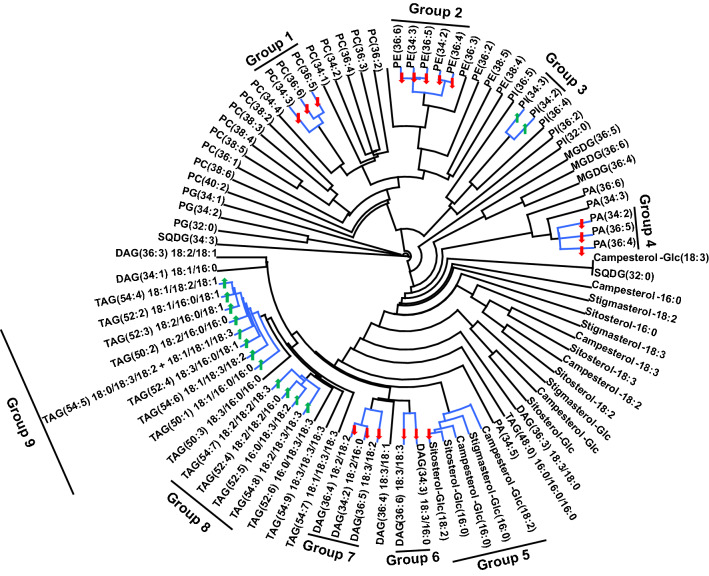
Dendrogram demonstrating co-occurring lipid groups in peanut anther. All 89 lipid species were clustered using a single-linkage hierarchical algorithm based on Spearman’s correlation coefficient, ρ. Lipid groups with ρ ≥ 0.90 are indicated by blue-colored bars on the dendrogram. The arrows on the dendrogram represent statistically significant changes in the level of each lipid (based on percentage of total signal) under heat stress (41/27 °C for 2018 and 38/26 °C for 2019; average day/night temperatures during the 17-days and 18-days treatment periods in 2018 and 2019, respectively) compared to ambient temperature (31/22 °C for 2018 and 28/22 °C for 2019). Green-colored upward arrows indicate significant increases and red-colored downward arrows indicate significant decreases in the levels of lipids. Data from 2 years (2018 and 2019) were pooled together for analysis. DAG, diacylglycerols; MGDG, monogalactosyldiacylglycerol; PA, phosphatidic acid; PC, phosphatidylcholine; PE, phosphatidylethanolamine; PG, phosphatidylglycerol; PI, phosphatidylinositol; SQDG, sulfoquinovosyldiacylglycerol; TAG, triacylglycerol. Lipid molecular species are identified as total acyl carbons: total double bonds. Acyl-chain composition is included after each DAG and TAG lipid species but does not indicate *sn* positions. CLUSTER 3.0 was used to determine clusters (Version 1.59, http://bonsai.hgc.jp/~mdehoon/software/cluster/software.htm).

A dendrogram can detect lipids experiencing coordinated metabolism based on the analysis of lipid co-occurrence^14,26^. Nine lipid groups were identified on the dendrogram with ρ ≥ 0.90 (i.e., each lipid in a group is correlated with at least one other lipid in the same group with ρ ≥ 0.90) (Fig. 7). Thus, the lipid groups included co-occurring lipid species. Group 1 included PC(36:5), PC(36:6), and PC(34:3), whereas group 2 included PE(34:3), PE(34:2), PE(36:6), PE(36:5), and PE(36:4). Of the aforementioned, PC(36:5), PC(36:6), PC(34:3), PE(34:3), PE(36:6), and PE(36:5) contain one or two 18:3 acyl chains. Groups 8 and 9 together included 12 TAG species. The clustering of these molecular species suggests that lipids in each group underwent coordinated metabolism, as the species increased or decreased together across individual samples subject to the two treatments. Groups 1 and 2 lipids decreased under HT, whereas TAGs in groups 8 and 9 increased. In addition, 11 of these lipids: PC(36:6), PC(36:5), PC(34:2), PE(36:5), PE(34:3), TAG(18:2/16:0/18:1) [i.e., TAG(52:3)], TAG(16:0/18:3/18:2) [i.e., TAG(52:5)], TAG(18:2/18:2/16:0) [i.e., TAG(52:4)], TAG(18:1/18:2/18:1) [i.e., TAG(54:4)], TAG(18:2/16:0/16:0) [i.e., TAG(50:2)], and TAG(18:1/18:3/18:2) [i.e., TAG(54:6)] were also included among the 15 most important lipids that differentiated HT from AT using the PLS-DA (Fig. 6b).

### Expression level changes occurring in the peanut *FAD* genes upon exposure to heat stress conditions

We hypothesized that observed differences in the lipid unsaturation levels among peanut genotypes in response to the variation in the growing temperatures might correspond with the expression levels of the peanut omega-6 (*FAD2* and *FAD6*) and omega-3 (*FAD3* and *FAD7*) desaturase genes. In the peanut genome, there exist three copies each of *FAD2* (*FAD2-1A*, *FAD2-1B*, and *FAD2-2*) and *FAD7 *(*FAD7-1A, FAD7-1B,* and *FAD7-2*) genes, four copies of *FAD3* gene (*FAD3-1*, *FAD3-2A*, *FAD3-2B*, and *FAD3-2C*), and one copy of *FAD6* gene^27,28^. Fatty acid desaturase 2 (oleate desaturase) converts 18:1 extraplastid-localized lipids to 18:2, FAD3 (linoleate desaturase) converts 18:2 extraplastid-localized lipids to 18:3, FAD6 converts 18:1 plastid-localized lipids into 18:2, and FAD7 converts 18:2 plastid-localized lipids to 18:3. *Fatty acid desaturase 2-1* and *FAD6* were reported earlier to exhibit higher expression in roots and leaves upon drought exposure^27^. The transcripts of *FAD2-2* were shown to over-accumulate in roots upon exposure to salt, drought, and ABA treatments, and in leaves upon exposure to drought treatment^27^. The transcripts of *FAD3-1* were shown to have enhanced accumulation in the salt-stressed roots and *FAD3-2* transcripts in drought and salt-stressed and ABA-treated roots^27^. Additionally, *FAD2-1*, *FAD2-2*, *FAD3-1*, and *FAD3-2 *genes were reported earlier to express in floral tissues^27,29^. On the contrary, the *FAD6*, *FAD7-1,* and *FAD7-2 *genes were expressed primarily in either vegetative tissues or seeds^27,30^. Given this prior knowledge, we set out to test the expression levels of the peanut *FAD2-1*, *FAD2-2*, *FAD3-1*, and *FAD3-2* genes in anthers of six peanut genotypes grown at AT and HT. Wynne (2.8 fold), Georgia 12Y (3.2 fold), and Tifguard (3.9 fold) showed increased expression (> 1.5-fold) of *FAD2-1 *under HT (Fig. 9). Additionally, Bailey showed increased expression of *FAD2-2 *(3.9 fold) and Georgia 12Y showed increased expression of *FAD3-2* (1.8 fold) under HT (Fig. 9). On the other hand, SPT 06-07 (1.7-fold) showed reduced accumulation of *FAD3-2* under HT (Fig. 9).

## Discussion

The decreases in chlorophyll index and increases in thylakoid membrane damage under heat stress demonstrate that the peanut genotypes evaluated in this study were significantly affected by heat stress at the leaf level (Fig. 1). The reproductive capacity of the genotypes was also affected by heat stress, which is evident from the 49 to 87% reduction in pollen viability (Fig. 1). The decreased pollen viability was clearly reflected in decreased pod number (15 to 55% reduction) under HT (Fig. 1). The above results show that heat stress significantly affected the physiology and reproductive capacity of the peanut genotypes, which led to reduced yield. However, the impact of HT differed among genotypes in terms of the above traits (chlorophyll index, thylakoid membrane damage, pollen viability, and pod number); Bailey, Wynne, and Sugg were most impacted, whereas SPT 06-07, Georgia 12Y, and Tifguard were least impacted.

PC and PE together make up the bulk of the lipidome under AT and HT treatments in peanut anthers (Fig. 2a). This finding is consistent with previous studies that report the predominance of PC and PE among polar lipids in non-photosynthetic plant tissues such as root, flower, seed, and pollen^15,25,31,32^. PC is the central intermediate in the synthesis of other glycerophospholipids, and both PC and PE are major structural components of cellular membranes^33,34^. In addition, both PC and PE fulfill other functions in plant growth and reproduction. PC is required for cell proliferation and differentiation^35^. Another group studied the effect of a double knockout of *aminoalcoholphosphotransferase 1 and 2* (*aapt1/2*), enzymes involved in PC synthesis, in *Arabidopsis thaliana* and found that the consequences were retarded growth and ~ 40% reduction in pollen viability compared to the wild type^36^. PE fills major roles in autophagy, cell division, and protein folding and other protein modifications^37,38^.

**Figure 8:**
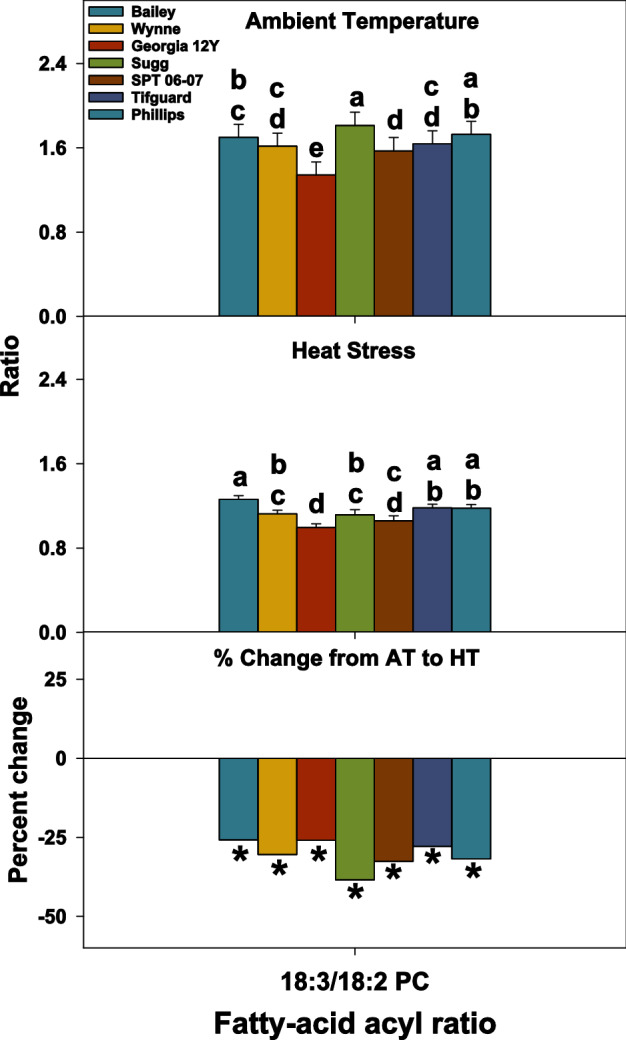
Changes in 18:3/18:2 phosphatidylcholine (PC) lipid ratio in peanut anthers in response to heat stress. Values shown are least-squares means. Error bars represent standard errors about the least-squares mean of 16 observations (2 years × 2 blocks × 4 replications) except for Sugg  and  SPT  06–07, which have 8 observations (1 year × 2 blocks × 4 replications). Least-squares means with different letters are significantly different according to Fisher’s least significant difference (LSD) test at α = 0.05. An asterisk (*) above bars indicate a significant difference at α = 0.05 between AT and HT for that genotype. AT, ambient temperature (31/22 °C for 2018 and 28/22 °C for 2019; average day/night temperatures during the 17-days and 18-days treatment periods in 2018 and 2019, respectively); HT, high temperature (41/27 °C for 2018 and 38/26 °C for 2019). Data from 2 years (2018 and 2019) were pooled together for analysis. Lipid molecular species are identified as total acyl carbons: total double bonds.

Though heat stress negatively impacted all examined peanut genotypes, it still triggered some cellular level mechanisms that could be acclimation to the stress, and the genotypes differed in their responses. Heat stress caused remodeling in the anther lipidome of peanut genotypes. For example, PC and PE species that contained 18:3 acyl chains (e.g., 34:3, 36:6, and 36:5) decreased under heat stress (Fig. 4). This led to a decrease in the unsaturation indices of PC and PE (Fig. 3). Fatty-acyl chains in plant membranes commonly have *cis* double bonds that reduce the degree of compaction in membranes by introducing bends in the acyl chains^39^. Heating also reduces membrane compactness. By decreasing the number of double bonds under high temperatures, plants can maintain optimal membrane compactness and consequentially optimal membrane fluidity and integrity. The reduction in phospholipid fatty acid unsaturation could be an acclimation mechanism under heat stress^40^. However, some genotypes were less responsive than others. For example, Bailey failed to significantly decrease the amounts of highly unsaturated species PC(34:3) and PC(36:5) under HT. Compared to Bailey, SPT 06-07 and Georgia 12Y both had significantly lower amounts of 34:3, 36:6, and 36:5 PC under HT (Fig. 4). Consequentially, both SPT 06-07 and Georgia 12Y had significantly lower unsaturation levels for PC compared to Bailey under HT, although the three genotypes had similar levels under AT (Fig. 3). Bailey had poor pollen viability and yield relative to that of Georgia 12Y and SPT 06-07, which indicates that changes in the anther lipidome might have influenced pollen performance and ultimately yield. Furthermore, all genotypes had a significant decrease in the ratios of 18:3/18:2 PC under HT (Fig. 8). SPT 06-07 had a greater decrease in the 18:3/18:2 PC ratio than Bailey (Fig. 8). Taken together, our results suggest that the decline in the level of lipid unsaturation by decreasing the relative amounts of the polyunsaturated fatty acid 18:3 is likely an acclimation mechanism to heat stress in peanut plants. Furthermore, genotypes that are more efficient in decreasing the lipid unsaturation levels by decreasing the polyunsaturated fatty acid amounts are relatively more tolerant to HT.

TAGs accounted for 5–13% of the total mass spectrometry signal from lipids of peanut genotypes under AT. The proportion of TAG substantially increased to 17–31% under HT (Fig. 2a). The percentage increase in TAG levels under HT was highest in the more heat-tolerant genotypes, SPT 06-07 and Georgia 12Y, and lowest in the least tolerant genotype, Bailey (Fig. 2a). When viewed as a ratio of TAG:PC, the differences among genotypes are even more striking with SPT 06-07 and Georgia 12Y having TAG:PC ratios under HT as 1.36 and 0.99, respectively, while in Bailey this ratio was 0.52 (Supplementary Fig. S4). Previous studies have reported that defects in TAG biosynthesis can result in sterile pollen^41^. Based on this, we hypothesize that peanut genotypes that can form more TAGs under HT have a higher level of thermotolerance.

The TAGs that were formed during HT contained a variety of molecular species but were least enriched in the most saturated (16:0/16:0/16:0) and the most unsaturated (18:3/18:3/18:3) molecular species (Fig. 5). The lack of increase in the TAG(16:0/16:0/16:0) species may be because a significant portion of high-melting 16:0 component remains in polar lipids in order to stabilize membranes at HT. The lack of much increase in TAG(18:3/18:3/18:3) in most genotypes at HT is in contrast to the large increases seen in TAG(18:3/18:3/18:3) in Arabidopsis seedlings and leaves and in wheat leaves^13,21–24^. Muller et al.^21,22^ reported that the accumulation of TAG species, particularly those with polyunsaturated acyl chains, is a heat-response mechanism. The TAG formed in peanut anthers under HT contained all the major fatty acids but was less enriched in 18:3 than in the other major fatty acids, in contrast to the results in Arabidopsis^21–24^. Essentially no TAG(18:3/18:3/18:3) was accumulated under HT in peanut anthers (Fig. 5). Besides the plant species and tissue differences, another difference between the Arabidopsis and peanut studies was the length of the heat treatment. The Arabidopsis studies subjected the plants to heat for 1 day or less, whereas the current study subjected the peanut plants to heat for 17 to 18 days. One possible explanation is that 18:3 removed from polar lipids is sequestered in TAG in the short term, but in the longer term, 18:3 is catabolized by beta oxidation. Newly synthesized fatty acids are less unsaturated (more 18:1), and in the longer-term heat treatment, the fatty acids in the polar and TAG pools equilibrate, with both pools having reduced unsaturation in HT compared to AT.

Using PLS-DA to determine the most important lipids differentiating the ambient (control) and heat stress treatments, polyunsaturated acyl containing PC, PE, PI (34:3, 36:6, and 36:5 species) and TAG species were ranked highly. This supports the notion that peanut plants responded to HT by lowering unsaturation levels of extraplastid-localized phospholipids and raising levels of TAG species (Figs. 3, 4, and 5).

We also conducted PLS-DA on wheat pollen lipid data generated by our previous research (see Supplementary Table S1 in Narayanan et al.^15^) to test if similar trends exist across species. This data set (Supplementary Table S1 in Narayanan et al.^15^) presents the amounts of 89 lipid species measured in pollen samples collected from Karl 92, a heat-susceptible wheat genotype, exposed to optimum temperature and high day and night temperature. The PLS-DA analyzed the relative importance of lipid species in differentiating the two treatments (optimum temperature and heat stress) (Supplementary Fig. S5). Like that of peanut anther, PLS-DA of wheat pollen classified PC(36:5), PC(34:3), PC(36:6), and PE(34:3) as the top four most important features (Supplementary Fig. S5). These results demonstrate that PC and PE species, and in particular the above four [PC(36:5), PC(34:3), PC(36:6) and PE(34:3)], considerably influence the heat-stress response in the reproductive tissues of peanut and wheat.

We further examined lipids experiencing coordinated metabolism under heat stress. This analysis revealed that PC(34:3), PC(36:5), and PC(36:6) formed a co-occurring lipid group (Group 1), which was downregulated under HT (Fig. 7). PE(34:3), PE(36:5), PE(36:6), PE(34:2), and PE(36:4) formed another co-occurring lipid group (Group 2), which was also downregulated under HT (Fig. 7). The decrease in Group 1 lipids under HT is most likely due to them containing one or more 18:3 acyl chains. Group 2 contained lipids that have 18:3 acyl chains (34:3, 36:6, and 36:5 PE) and lipids that do not (34:2 and 36:4 PE). Therefore, the downregulation of Group 2 lipids is most likely coordinated by their PE headgroup, which is known to promote the formation of non-bilayer phases, along with the presence of 18:3 acyl chains.

**Figure 9:**
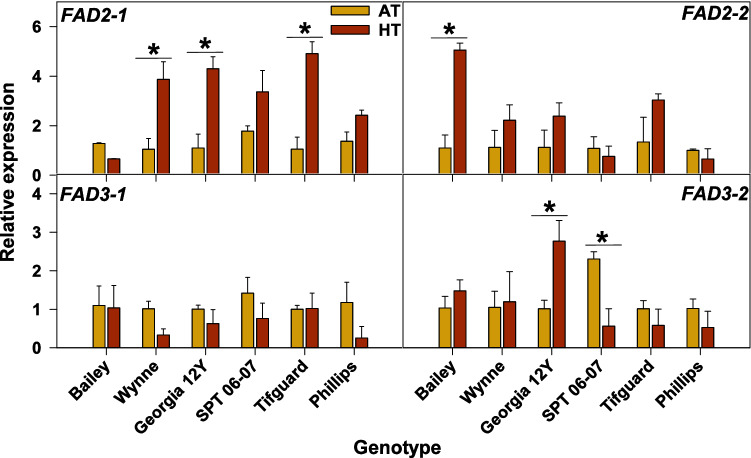
Changes in expression levels of the Fatty Acid Desaturase (FAD) genes: *FAD2-1*, *FAD2-2*, *FAD3-1*, and *FAD3-2* under heat stress in the anther samples of six peanut cultivars grown under field conditions in 2019. RT-PCR was performed using gene-specific primers, and in all cases, gene expression results are normalized to the expression of the housekeeping gene *Actin-7* (see Supplementary Table S4). For qRT-PCR analysis, three biological and two technical replicates were used. Asterisk (*) marks indicate significant differences (> 1.5-fold) in the expression level of the *FAD* genes observed under two treatments. The error bars signify the standard error. AT, ambient temperature (28/22 °C; average day/night temperatures during the 18-days treatment period); HT, high temperature (38/26 °C).

Our earlier research on soybean demonstrated that a decrease in the level of lipids containing 18:3 acyl chains (linolenic acid) under heat stress in the tolerant genotype is a likely consequence of the reduced activity of the *FAD3A* and *FAD3B* genes and contributes to its heat tolerance^16^. In line with this study, transgenic silencing of the endoplasmic reticulum (ER)-localized fatty acid desaturase gene, *FAD3*, and the chloroplast-localized desaturase gene, *FAD7*, was shown to enhance tolerance of tomato plants to heat stress^42–44^. In the present study, Georgia 12Y showed 1.8-fold higher expression of *FAD3-2*, while SPT 06-07 showed 1.7-fold reduced expression of *FAD-3-2*, whereas Bailey did not show any change for both *FAD3-1* and *FAD3-2* (Fig. 9). These results corresponded with the lipid profiling data of both SPT 06-07 and Bailey and their respective field performances (i.e., larger reduction in the content of 18:3 lipids and a better field performance for SPT 06-07 than Bailey) (Figs. 1, 2, 3, 4 and 9). Since Georgia 12Y exhibited a contrasting *FAD3-2* expression pattern under heat stress, it appears that this genotype operates a different heat tolerance mechanism.

Since the *FAD3* genes encode linoleate desaturase, which converts 18:2 fatty acids to 18:3, a decline in the *FAD3* expression level in SPT 06-07 reduced the content of 18:3 lipids. It corroborates with the earlier findings in soybean, where the correspondence between heat-induced suppression of *FAD3A, FAD3B*, and *FAD3C* expression and a subsequent reduction in the seed linolenic acid (18:3) content were observed^45^. The peanut *FAD3-2* gene corresponds with the soybean *FAD3C* genes (EU678358.1; 84% sequence identity). Hence, it suggests that this mechanism of heat tolerance via reducing the *FAD3* expression level is conserved across genera. More evidence supporting this hypothesis could be gathered by monitoring differences in the accumulation of encoded proteins and their lipid products and/or studying the effects of these changes on membrane composition and functioning. Post-translational regulation of the FADs and the transcriptional regulation by feedback or other mechanisms could be other possibilities, which will be tested in future studies.

**Figure 10:**
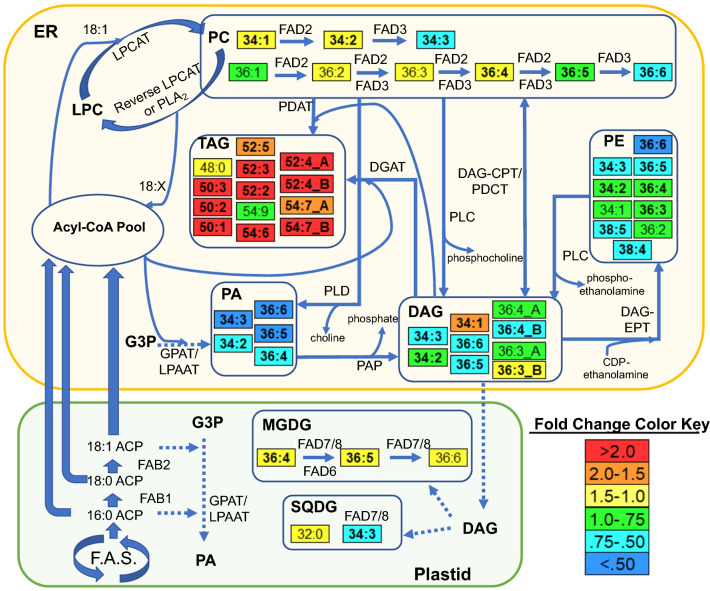
A lipid metabolic pathway map based on previously published information and results from the present study^14,23,24,59–62^. Dashed arrows indicate multi-step conversions. Lipid molecular species are identified as total acyl carbons: total double bonds. The fold change was calculated as the value of the lipid species under HT/the corresponding value under AT. Bolded lipids are significantly increased or decreased at α = 0.05 (Supplementary Table S3). ACP, acyl carrier protein; CoA, coenzyme A; DAG, diacylglycerol; DAG-CPT, CDP-choline:diacylglycerol cholinephosphotransferase; DAG-EPT, CDP-ethanolamine:diacylglycerol cholinephosphotransferase; DGAT, acyl-CoA:diacylglycerol acyltransferase; ER, endoplasmic reticulum; FAB, fatty acid biosynthesis; FAD, fatty acid desaturase; F.A.S., fatty acid synthesis; G3P, glycerol-3-phosphate; GPAT, glycerol-3-phosphate acyltransferase; LPAAT, lysophosphatidic acid acyltransferase; LPC, lysophosphatidylcholine; LPCAT, lysophosphatidylcholine acyltransferase; MGDG, monogalactosyldiacylglycerol; PA, phosphatidic acid; PAP, phosphatidic acid phosphatase; PC, phosphatidylcholine; PDAT, phospholipid:diacylglycerol acyltransferase; PDCT, phosphatidylcholine:diacylglycerol cholinephosphotransferase; PE, phosphatidylethanolamine; PG, phosphatidylglycerol; PI, phosphatidylinositol; PLA_2_, phospholipase A_2_; PLC, phospholipase C; PLD, phospholipase D; SQDG, sulfoquinovosyldiacylglycerol; TAG, triacylglycerol. Lipids with ‘A’ or ‘B’ have different acyl chain compositions (Supplementary Table S3), i.e., DAG(36:3)_A is DAG(18:3/18:0); DAG(36:3)_B is DAG(18:2/18:1); TAG(52:4)_A is TAG(18:3/16:0/18:1); TAG(52:4)_B is TAG(18:2/18:2/16:0); TAG(54:7)_A is TAG(18:2/18:2/18:3); TAG(54:7)_B is TAG(18:1/18:3/18:3) (Acyl-chain composition included after each DAG and TAG lipid species does not indicate *sn* positions).

Our results provide novel information about heat-stress responses in peanut anthers, which can be placed within the broader context of plant lipid metabolism as shown in Fig. 10. Lipid biosynthesis in plants follow a general route where fatty acids are synthesized in the plastid, exported to the ER, and undergo further modifications when attached to PC (Fig. 10). Modified fatty acids can return to the acyl-CoA pool via the reverse reaction of lysophosphatidylcholine acyl transferase (LPCAT) or via phospholipase A_2_ (PLA_2_); be transferred to DAG lipids by removal of the entire phosphoryl headgroup via phospholipase C (PLC), the removal of the PC headgroup by phospholipase D (PLD) producing PA followed by removal of the remaining phosphate by phosphatidic acid phosphatase (PAP), or incorporation of modified fatty acids from the acyl-CoA pool through the Kennedy pathway; be transferred to PE lipids by first converting to DAG lipids as previously mentioned followed by transfer of an ethanolamine headgroup by CDP-ethanolamine:diacylglycerol cholinephosphotranserase (DAG-EPT); or be incorporated into TAG via phospholipid:diacylglycerol acyltransferase (PDAT) or acyl-CoA:diacylglycerol acyltransferase (DGAT) (Fig. 10). In the presented study, the fatty acyl chains sequestered from ER-localized lipids (e.g., PC) seem to have been redirected into TAG. The differences in heat stress responses among genotypes were associated with the FAD3 mediated step in the lipid pathway (i.e., tolerance was associated with reduced *FAD3* expression leading to reduced unsaturation levels of ER lipids).

In summary, peanut anthers predominantly contained PC, PE, and TAG lipid species under AT and HT conditions. Heat stress caused decreases in the unsaturation indices of both PC and PE, which was primarily due to decreases in the highly-unsaturated lipid species that contained linolenic (18:3) acid. 36:6, 36:5, and 34:3 PC and PE(34:3) (all contain 18:3 fatty acid and decreased under HT) were the most important lipids that differentiated HT from AT. PC(34:3), PC(36:5), and PC(36:6) and PE(34:3), PE(34:2), PE(36:6), PE(36:5), and PE(36:4) underwent coordinated metabolism and formed co-occurring lipid groups, which were down-regulated under heat stress. TAGs increased under HT indicating their role in maintaining pollen viability and serving as a recycling reservoir for the fatty acyl chains sequestered from the membrane lipids. The decline in the level of lipid unsaturation by decreasing the polyunsaturated fatty acid, linolenic (18:3) acid, through the decrease in *FAD3* expression, is likely an acclimation mechanism to heat stress in peanut plants. Furthermore, genotypes that are more efficient in decreasing the *FAD3* expression and thus, the lipid unsaturation levels will be relatively more tolerant to HT.

## Conclusions

In this study, we investigated the changes in the anther lipidome of seven peanut genotypes under heat stress conditions. Peanut anthers underwent significant lipid remodeling under HT likely to maintain optimal membrane fluidity and prevent phase transition into non-bilayer phases. Our results provide novel information about HT responses in peanut. Considering that the flowering stage is highly sensitive to heat stress in peanut, this study serves as a useful platform to further probe acclimation mechanisms to HT in peanut reproductive tissue and identify lipid biomarkers and corresponding molecular markers that will aid in breeding heat-tolerant varieties with better pollen performance.

## Materials and methods

### Plant material

The peanut genotypes evaluated in this study were Bailey, Wynne, Georgia-12Y, Sugg, Tifguard, Phillips, and SPT 06-07. All of them except SPT 06-07 are released varieties, which are currently under cultivation in the Virginia-Carolina region in the USA. These genotypes were selected for the present study based on their differential responses to heat or drought stress^46–49^ and personal correspondence with peanut breeders/specialists, Balota M. and Isleib T. G. Bailey and Phillips were found to have reduced membrane injury during heat stress^46^. Georgia-12Y has drought tolerance based on gross dollar value return per hectare^49^. Tifguard has drought tolerance based on multiple physiological traits (e.g., chlorophyll fluorescence, chlorophyll index, and canopy temperature depression) and pod yield^46^. Sugg and Wynne have large pod size, which makes them susceptible to drought stress^48^. SPT 06-07 has been identified as potentially heat tolerant based on metabolic (increased galactinol, reduced hydroxyproline, and higher saturated vs. unsaturated fatty acid ratio) and physiological (lower membrane injury) traits^47^.

### Plant husbandry and heat stress treatment

Peanut plants of genotypes Bailey, Wynne, Georgia 12Y, Sugg, Tifguard, and Phillips were grown under field conditions at the Clemson University Simpson Research and Education Center, Pendleton, SC, USA (34°38′51.4″N, 82°43′41.1″W and 260 m a.s.l.) during the 2018 cropping season (June to November). The experiment was repeated in 2019 (June to November) with the same genotypes except Sugg, which was substituted by SPT 06-07. Sugg made way for SPT 06-07 in 2019 due to space limitation in the heat tents and Sugg having the least contrasting results in 2018. The characteristics of the experimental site are given in Supplementary Table S1. Field operations followed the recommendations in the Clemson University Peanut Money Maker Guide^48^; details are given in Supplementary Table S1. Weeds were controlled through pre-emergent application of herbicides in 2018 and by hand weeding in 2019 (Supplementary Table S1). Additionally, hand-weeding was performed whenever needed in both years. All genotypes were sown in single-row plots (see Supplementary Table S1 for details). The treatment design was a two-factor factorial based on temperature (two levels- AT and HT) and genotype (six genotypes as six levels) resulting in 12 treatment combinations. The experiment design was a split plot (see field map in Supplementary Fig. S1). Temperature was the whole plot factor with two replications per level arranged as a randomized complete block design. Genotype was the split-plot factor with four replications per level arranged as a randomized complete block design within the whole-plot blocks. All plots were maintained as rain-fed throughout the cropping season. No pest or pathogen problems were observed for the duration of the cropping season. Air temperature was monitored every 15 min at the plant canopy level (~ 65 cm from the soil surface) using HOBO data loggers (Onset Computer Corporation, Bourne, MA, USA) from planting through final harvest at maturity (Supplementary Figs. S2, S3).

In both years, heat stress treatment was initiated when the last genotype reached anthesis in both treatments in each block. This was determined by first tagging 10 consecutive plants within each plot and then tagging each of them again when they reached anthesis. Genotypes per treatment per block were considered to have reached anthesis when 50% of the 10 tagged plants in one of the four plots (four replications) reached anthesis^50^. The first and last genotype to reach anthesis differed by 7 days in both years. Heat stress treatment was imposed for 17 days and 18 days in 2018 and 2019, respectively, using two heat tents (Supplementary Fig. S1). Each heat tent was 4.9 m wide, 13.7 m long, and 3.5 m high. Heat tents were constructed with a galvanized steel framework covered with a removable 6 mil-clear, greenhouse plastic film (Supplementary Fig. S1), which transmits 91% of the incoming solar radiation. The heat tents increase the inside temperature by radiative heating (greenhouse effect). Therefore, the temperature inside the heat tents is dependent on the intensity of solar radiation and the outside ambient temperature. The observed air temperatures inside the heat tents at 65 cm from the soil surface (plant canopy level) are given in Supplementary Figs. S2 and S3. The average daytime (06:45 to 20:30) temperatures in 2018 during the 17-days treatment period were 41 °C inside the heat tents and 31 °C outside the heat tents, and the temperatures in 2019 during the 18-d treatment period were 38 °C inside the heat tents and 28 °C outside (Supplementary Figs. S2, S3). Average night-time temperatures during the treatment period in 2018 were 27 °C inside the heat tents and 22 °C outside the heat tents, and the night-time temperatures in 2019 were 26 °C inside the heat tents and 22 °C outside (Supplementary Figs. S2, S3). Both heat tents maintained a similar amount of photosynthetically active radiation (PAR) inside, as compared to ambient conditions [~ 2000 μmol m^−2^ s^−1^] around solar noon; measured using a PAR sensor (Quantum Light 6 Sensor, Spectrum Technologies, Aurora, IL, USA). After the stress treatment period (17 days in 2018 and 18 days in 2019), the greenhouse film covering each heat tent was removed so that all plots remained under AT until final harvest at maturity. In 2018, a total of 416 mm of rainfall occurred during the growing season and 75 mm during the treatment period (Supplementary Fig. S2). The total rainfall in 2019 was 444 mm during the growing season and 69 mm during the treatment period (Supplementary Fig. S3). Rain was not able to enter the heat tents while covered with the greenhouse film.

### Physiological and yield traits

In 2018 and 2019, a representative plant was tagged in each plot during the vegetative stage for collecting data on physiological traits. A total of eight plants were tagged per genotype per treatment (AT and HT) (two blocks × four replications per block) in each year for this purpose. Chlorophyll index and fluorescence were measured on the 16th day of the treatment period in both years. Chlorophyll index, which is an instantaneous measurement of “greenness” of leaves, affects leaf photosynthetic capacity. It is often used as an indicator of abiotic stress impacts on plants. We measured chlorophyll index of peanut plants using a self-calibrating chlorophyll meter [Soil Plant Analytical Device (SPAD) 502; Spectrum Technologies, Aurora, IL, USA]. A single measurement was taken on each of the four leaflets of the youngest-fully-expanded leaf, and readings were averaged for each plant. Chlorophyll *a* fluorescence parameters were measured on one of the two top leaflets of the youngest-fully-expanded leaf for each plant using a modulated fluorometer (OS30p+ ; Opti-Sciences, Hudson, NH, USA). Leaves were dark-adapted for 45 min before measuring minimum fluorescence (Fo) and maximum fluorescence (Fm). A ratio of Fo to Fm represents thylakoid membrane damage^51^.

Pollen viability was tested using 2% (w/v) triphenyl tetrazolium chloride (TTC) stain^8,52^. To extract pollen grains, freshly opened flowers were collected between 08:00 and 11:00 h for blocks 1 and 2 on the 14th and 15th days, respectively, of the treatment period in 2018. Similarly, flowers were collected for blocks 2 and 1 on the 10th and 11th days, respectively, of the treatment period in 2019. Flowers were collected from eight tagged plants per genotype per treatment. Pollen grains were extracted, spread on a microscope slide, and stained using TTC. The TTC stains the viable pollen grains with reddish-purple color due to the formation of insoluble formazan. The non-viable pollen grains remain as transparent (colorless). Pollen grains were observed using a compound light microscope (Leica DM500, Leica Microsystems, Buffalo Grove, IL, USA), and the number of pollen grains stained were recorded. Pollen viability was calculated as the ratio of viable pollen grains to total number of pollen grains and expressed as a %.

At harvest maturity (growth stage, R8), plants from 1-m row length were harvested from each plot in 2018. Harvested plants were dried down, and pods were separated using a XH-5000 Dry and Wet Type Peanut Picking Machinery (Henan Xuanhua Imp. & Exp. Trading Co., Ltd., Henan, China). In 2019, plants were harvested from 1-m row length, dried down, and pods were separated by hand. Pod number was counted and expressed as the number of pods per 1-m row length in both years.

### Anther collection and lipid extraction

To extract anthers, freshly opened flowers were collected from eight tagged plants per genotype per treatment in both years. Flowers were collected between 07:00 and 11:00 on the 13th day of the treatment period in 2018 from both treatments (AT and HT). In 2019, flowers were collected during the same time period on the 9th day of the treatment period from both treatments. Anthers from each flower were collected and transferred immediately into 3 mL of isopropanol with 0.01% butylated hydroxytoluene that was pre-heated for 15 min at 75 °C in a 30-mL glass test tube with a Teflon-lined screw-cap (DWK Life Sciences L.L.C., Millville, NJ, USA). Once anthers were in the isopropanol, they were kept at 75 °C for another 15 min to deactivate lipid-hydrolyzing enzymes. Afterwards, each glass tube with anthers was cooled to room temperature and brought to the laboratory for lipid extraction as previously described^13,15^. In the laboratory, 1.5 mL of chloroform and 0.6 mL of HLPC-grade water were added to each glass tube. Then, the glass tubes were stored at − 80 °C (pause point). For further analysis, the glass tubes stored at − 80 °C were brought out, allowed to reach room temperature, and shaken on an orbital shaker for 1 h at room temperature. Afterwards, the lipid extract in isopropanol, butylated hydroxytoluene, chloroform, and water from each glass tube was transferred using a Pasteur pipette to a new glass tube, while leaving the anthers in the original tube. To the glass tubes with anthers, 4 mL of chloroform:methanol (2:1) with 0.01% butylated hydroxytoluene was added. Then, the glass tubes containing anthers were shaken overnight on an orbital shaker at room temperature, followed by transfer of the solvent, which contains the lipid extract, into the tube containing the first extract. The addition of chloroform:methanol with 0.01% butylated hydroxytoluene, followed by overnight shaking, and then transfer of the solvent into the tube containing the first extract were carried out four times. After that, the solvent in lipid extract was evaporated using an N-EVAP 112 nitrogen evaporator (Organomation Associates, Inc., Berlin, MA, USA). Afterwards, the lipid extract was dissolved in 1 mL of chloroform and transferred to a 2-mL clear-glass vial with Teflon-lined screw-cap (DWK Life Sciences LLC., Millville, NJ, USA). These vials were stored at − 80 °C until preparation for shipping to the Kansas Lipidomics Research Center (KLRC) for lipid profiling. Before shipping, the solvent (chloroform) was again evaporated from the 2-mL vials using the N-EVAP 112 nitrogen evaporator. After that, the vials containing the lipid extracts were transported to KLRC over two days with dry ice. At KLRC, the lipid extracts were again dissolved in 1 mL of chloroform and were used for lipid profiling (described below). The anthers in the glass tubes were dried overnight at 105 °C, and their dry weights were measured to estimate lipid content on a dry weight basis. Anther dry weights were determined using a microbalance (AX26, Mettler Toledo, LLC, Columbus, OH, USA) which had a detection limit of 2 μg.

### Lipid profiling using an electrospray ionization-triple quadrupole mass spectrometry

In 2018 and 2019, lipid profiling was carried out using an automated electrospray ionization-tandem mass spectrometry approach, as previously described^13^. Precursor or neutral loss scanning was used to identify lipid molecular species. Lipid species in each headgroup class were quantified in comparison to internal standards, as previously described^13^. Identical samples of a quality-control pool, prepared by combining aliquots from each lipid sample, were analyzed recurrently among the experimental samples; the resulting data were used to calculate the coefficient of variation (CoV) of each analyte. The lipid values were calculated as normalized intensity per milligrams of anther dry weight, where a value of one is the intensity of 1 nmol of internal standard. The lipid values were converted to percentage of total normalized signal, as presented in Supplementary Table S2. Lipid analytes for which (1) the amount (normalized mass spectral signal) per milligrams of anther dry weight was less than the limit of detection (0.00005 nmol) or (2) the coefficient of variation (standard deviation divided by mean of the amount of the analyte in the quality-control samples) greater than 0.3 in either year were removed from the data set in order to maintain data quality.

### Lipid unsaturation index

Unsaturation index reflects the number of double bonds in a lipid, such that lipids with higher unsaturation index have more double bonds (degree of unsaturation). The unsaturation index for a lipid molecular species was calculated as the average number of double bonds per acyl chain, which is the number of double bonds in the lipid molecular species divided by the number of acyl chains. The unsaturation index of a lipid headgroup class was calculated as^13,15^:

$$\frac{\sum (\text{unsaturation indices of individual lipid molecular species in the class } \times \text{amount of each species})}{\sum \text{amount of all lipid molecular species in the class}}$$

### Anther collection for gene expression analysis, RNA extraction, and RT-qPCR

On the 12th day of the treatment period in 2019, anthers were collected between 08:00 and 12:00 from all plots. An open flower was collected from two different plants per plot for anther collection. Anthers from each flower were placed inside a sterile 2-ml microcentrifuge tube that was pre-chilled in liquid nitrogen. The microcentrifuge tube with anthers was immediately dropped inside a dewar that contained liquid nitrogen. The anthers in liquid nitrogen were transferred to a − 80 °C freezer upon returning to the lab and stored there until RNA extraction and the real-time quantitative PCR (RT-qPCR).

Total RNA was extracted from the anthers using the E.Z.N.A. HP Total RNA Kit (Omega Bio-tek, Inc., Norcross, GA) following the manufacturer’s instructions, including an on-column DNase step to avoid genomic DNA contamination. RNA was quantified using an ND-1000 spectrophotometer (NanoDrop, Wilmington, DE) and converted to cDNA using RevertAid First Strand cDNA Synthesis Kit (Thermo Fisher Scientific Inc., Waltham, MA) following the manufacturer’s recommendations. About 500 ng RNA was converted to cDNA.

The expression patterns of the *FAD2-1*, *FAD2-2*, *FAD3-1*, and *FAD3-2* genes were studied by the RT-qPCR analysis using the iTaq Universal SYBR Green Supermix chemistry on iCycler iQ from BioRAD following^16^. PCR primers for the peanut *FAD2-1*,* FAD2-2*,* FAD3-1*, and *FAD3-2* genes and the *Actin-7* gene (used as internal controls) were designed from cDNA sequences specifically to span the exon–intron junction to avoid amplification from the genomic DNA and are listed in Supplementary Table S4. The mRNA levels of the genes of interest were normalized to *Actin-7* using the Delta-Delta-CT (DDCT) method^53^. The transcript levels were expressed as a fold difference in the expression level of the target gene (normalized to *Actin-7*) in each genotype grown under AT and HT. Expression values for each genotype under control (AT) and heat treatment (HT) is an average of 12 values (i.e., three biological replicates × 2 technical replicates × 2 replicated plots).

### Data analyses

Analysis of variance and estimation of least-squares means and standard errors were carried out using the GLIMMIX procedure in SAS (Version 9.4, SAS Institute). Treatment and genotype were fixed effects and year and block were random effects. Separation of least-squares means was conducted based on Fisher’s least significant difference (LSD) test (α = 0.05) using the LSMEANS option in the GLIMMIX procedure. Utilities of the MetaboAnalyst web server (metaboanalyst.ca) were used to perform a PLS-DA^54^. Data scaling or normalization was not performed before PLS-DA analysis.

Creation of the dendrogram began with uploading lipid data (Supplementary Table S2) to CLUSTER 3.0 (Version 1.59, Open source software, Human Genome Center, The Institute of Medical Science, The University of Tokyo, Tokyo, Japan, http://bonsai.hgc.jp/~mdehoon/software/cluster/software.htm) to identify lipid groups^55^. The lipid groups were generated in CLUSTER 3.0 using a single-linkage hierarchical algorithm based on Spearman’s correlation coefficient. The clustering outputs (.gtr and .cdt files) generated by CLUSTER 3.0. were converted to NEWICK format (.nwk file) using a Python script written by Haibao Tang (J. Craig Venter Institute, Rockville, MD, USA). The script is accessible at the following link: https://github.com/tanghaibao/treecut/blob/master/scripts/eisen_to_newick.py. The NEWICK file was uploaded to Dendroscope to create the dendrogram, which was further modified in terms of color^56,57^. The clustering output from CLUSTER 3.0 (.cdt file) was viewed using Java Treeview (Open source software, available at http://jtreeview.sourceforge.net) for identifying ρ of lipid clusters (groups)^58^.

## Supplementary Information

Supplementary Figures.

Supplementary Tables.

## Data Availability

The lipid data generated or analyzed during this study are included in this published article and its Supplementary Table S2. The gene expression data generated and/or analyzed in the current study will be made available to the requestor upon reasonable request.
