# Supplementary material for: Heat stress elicits remodeling in the anther lipidome of peanut
Source: Sci Rep. 2020 Dec 17;10:22163. doi: 10.1038/s41598-020-78695-3 (PMC7747596; doi:10.1038/s41598-020-78695-3)
Supplement: Supplementary file 1 — Supplementary Figures. [file 41598_2020_78695_MOESM1_ESM.docx]

**Heat Stress Elicits Remodeling in the Anther Lipidome of Peanut**

**Zolian Sang Zoong Lwe^1^, Ruth Welti^2^, Dan Anco^1,3^, Salman Naveed^1,4^, Sachin Rustgi^1,4^, Sruthi Narayanan^1*^**

^1^Department of Plant and Environmental Sciences, Clemson University, Clemson, SC, USA

^2^Division of Biology, Kansas State University, Manhattan, KS, USA

^3^Edisto Research & Education Center, Clemson University, Blackville, SC, USA

^4^Pee Dee Research & Education Center, Clemson University, Florence, SC, USA

^*^Corresponding author: [skutty@clemson.edu](mailto:skutty@clemson.edu) (SN)


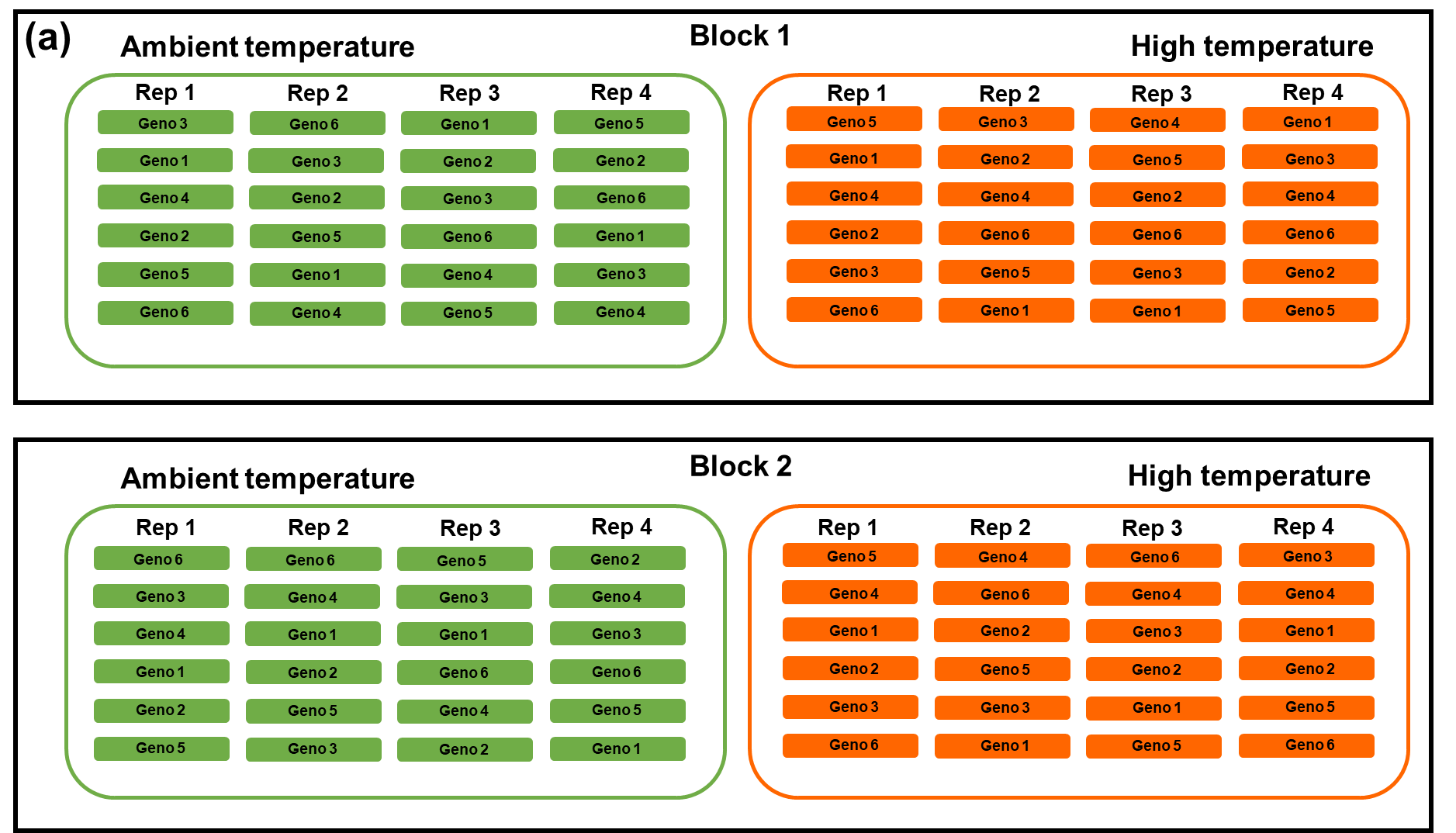


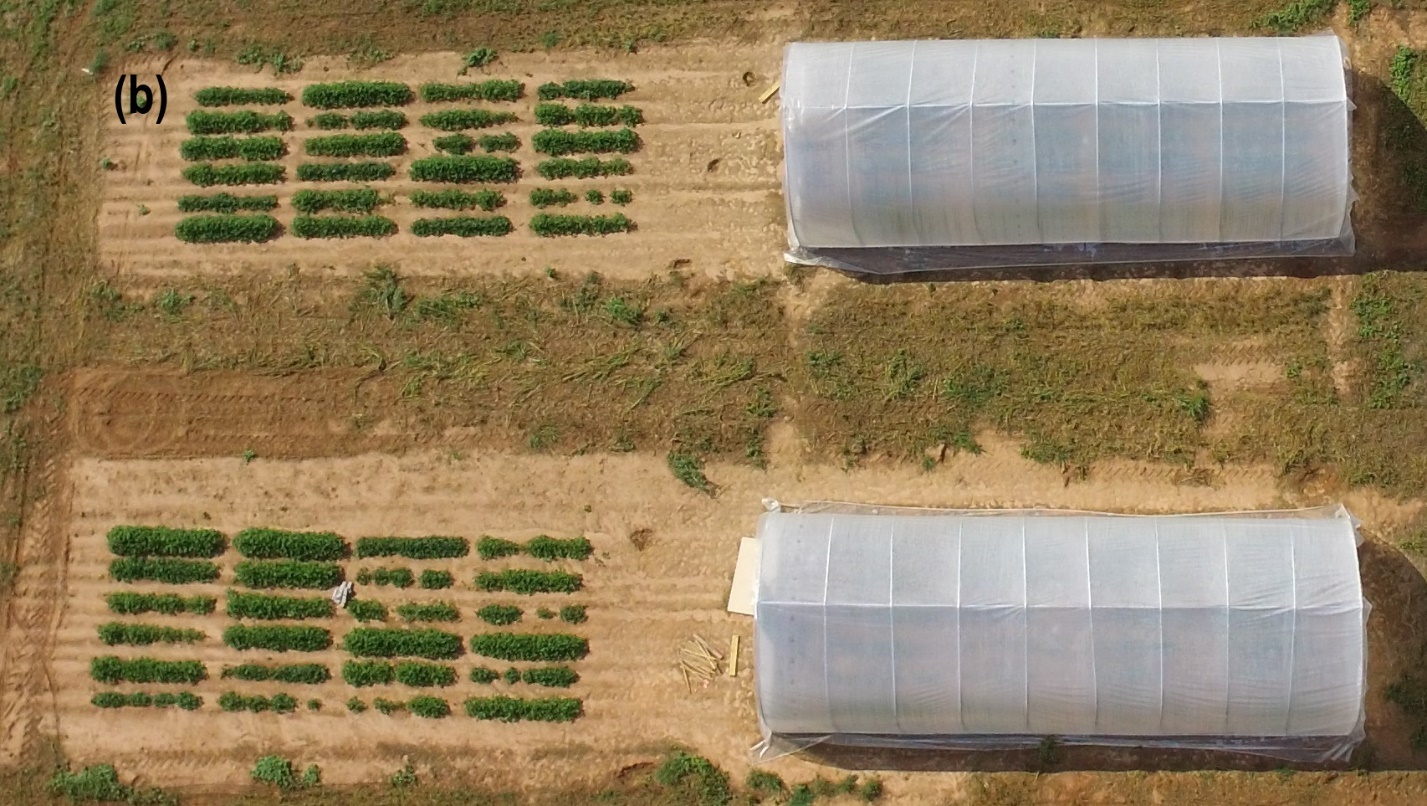


**Figure S1.** Field map (**a**) and a photograph taken during the treatment period (**b**). Experimental design was a randomized-complete-block design with a split-plot treatment structure. There were two blocks, each containing a section maintained under ambient temperature [control (31/22°C for 2018 and 28/22°C for 2019; average day/night temperatures during the 17- d and 18-d treatment periods in 2018 and 2019, respectively)] and another one designated for high temperature treatment [heat stress (41/27°C for 2018 and 38/26°C for 2019)], i.e., in the heat tent. Each section included four replications and each replication had a row of each of the six peanut genotypes. Genotypes Bailey, Wynne, Georgia 12Y, Sugg, Tifguard, and Phillips were grown in 2018. The same genotypes were used in 2019 except that Sugg was substituted by SPT 06-07. Geno, genotype.

**
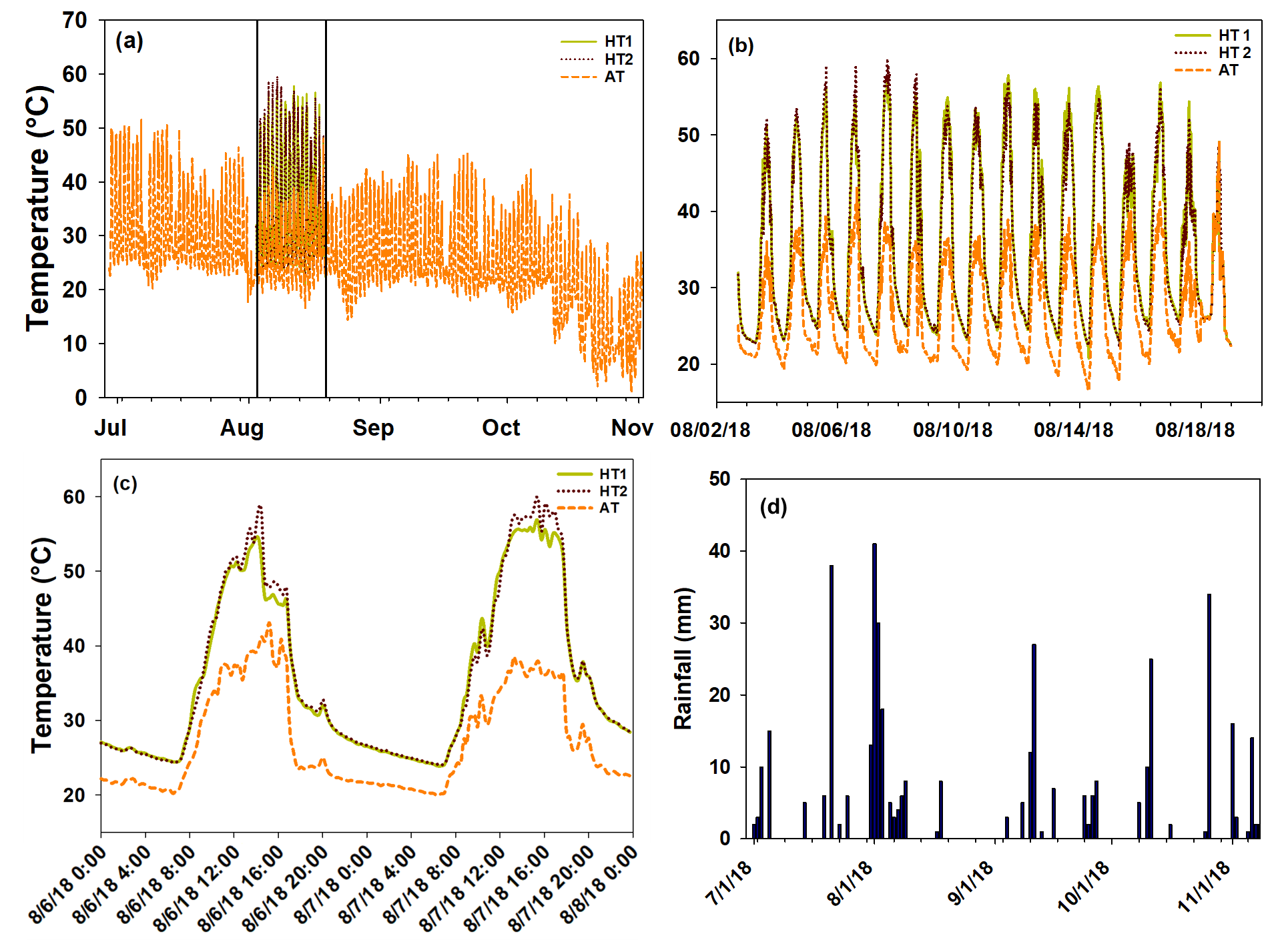
**

**Figure S2.** Air temperature (**a**-**c**) and rainfall (**d**) data during the 2018 peanut-growing season (29 June 2018 to 14 November 2018) and/or treatment period (02 August 2018 to 17 August 2018) at Pendleton, SC, USA. Panel ‘**a**’ presents the air temperature data for heat tent 1 (HT1), heat tent 2 (HT2), and open field (ambient temperature, AT) over the peanut growing season. Panel ‘**b**’ presents a closer look of the same data (i.e., air temperature for HT1, HT2, and AT) during the treatment period, and panel ‘**c**’ presents a further closer look of the same data over two days within the treatment period. The treatment period in panel ‘**a**’ is demarcated by black lines. Air temperature was measured every 15 min at the plant canopy level (~65 cm from the soil surface) using HOBO data loggers (Onset Computer Corporation, Bourne, MA, USA). As demonstrated by above figures, temperature inside both heat tents were essentially the same.


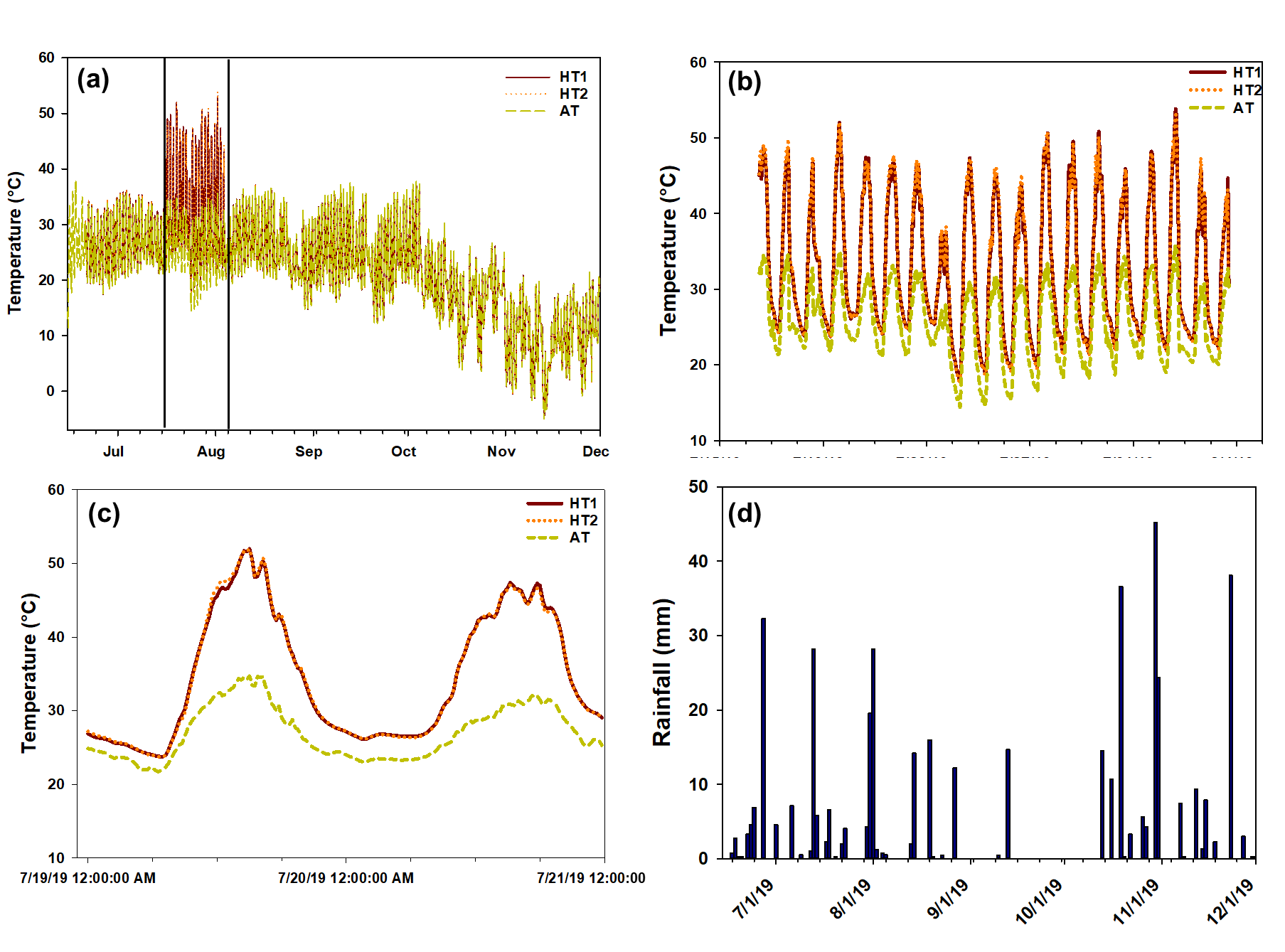


**Figure S3.** Air temperature (**a**-**c**) and rainfall (**d**) data during the 2019 peanut-growing season (14 June 2019 to 30 November 2019) and/or treatment period (16 July 2019 to 03 August 2019) at Pendleton, SC, USA. Panel ‘**a**’ presents the air temperature data for heat tent 1 (HT1), heat tent 2 (HT2), and open field (ambient temperature, AT) over the peanut growing season. Panel ‘**b**’ presents a closer look of the same data (i.e., air temperature for HT1, HT2, and AT) during the treatment period, and panel ‘**c**’ presents a further closer look of the same data over two days within the treatment period. The treatment period in panel ‘**a**’ is demarcated by black lines. Air temperature was measured every 15 min at the plant canopy level (~65 cm from the soil surface) using HOBO data loggers (Onset Computer Corporation, Bourne, MA, USA). As demonstrated by above figures, temperature inside both heat tents were essentially the same.

**Figure S4.** Changes in triacylglycerol:phosphatidylcholine (TAG:PC) ratio in peanut anthers in response to heat stress. Values shown are least-squares means. Error bars represent standard errors about the least-squares mean of 16 observations (2 years x 2 blocks x 4 replications) except for Sugg and SPT 06-07, which have 8 observations (1 year x 2 blocks x 4 replications). Least-squares means with different letters are significantly different according to Fisher’s least significant difference (LSD) test at α = 0.05. AT, ambient temperature (31/22°C for 2018 and 28/22°C for 2019; average day/night temperatures during the 17- d and 18-d treatment periods in 2018 and 2019, respectively); HT, high temperature (41/27°C for 2018 and 38/26°C for 2019). Data from two years (2018 and 2019) were pooled together for analysis.

**
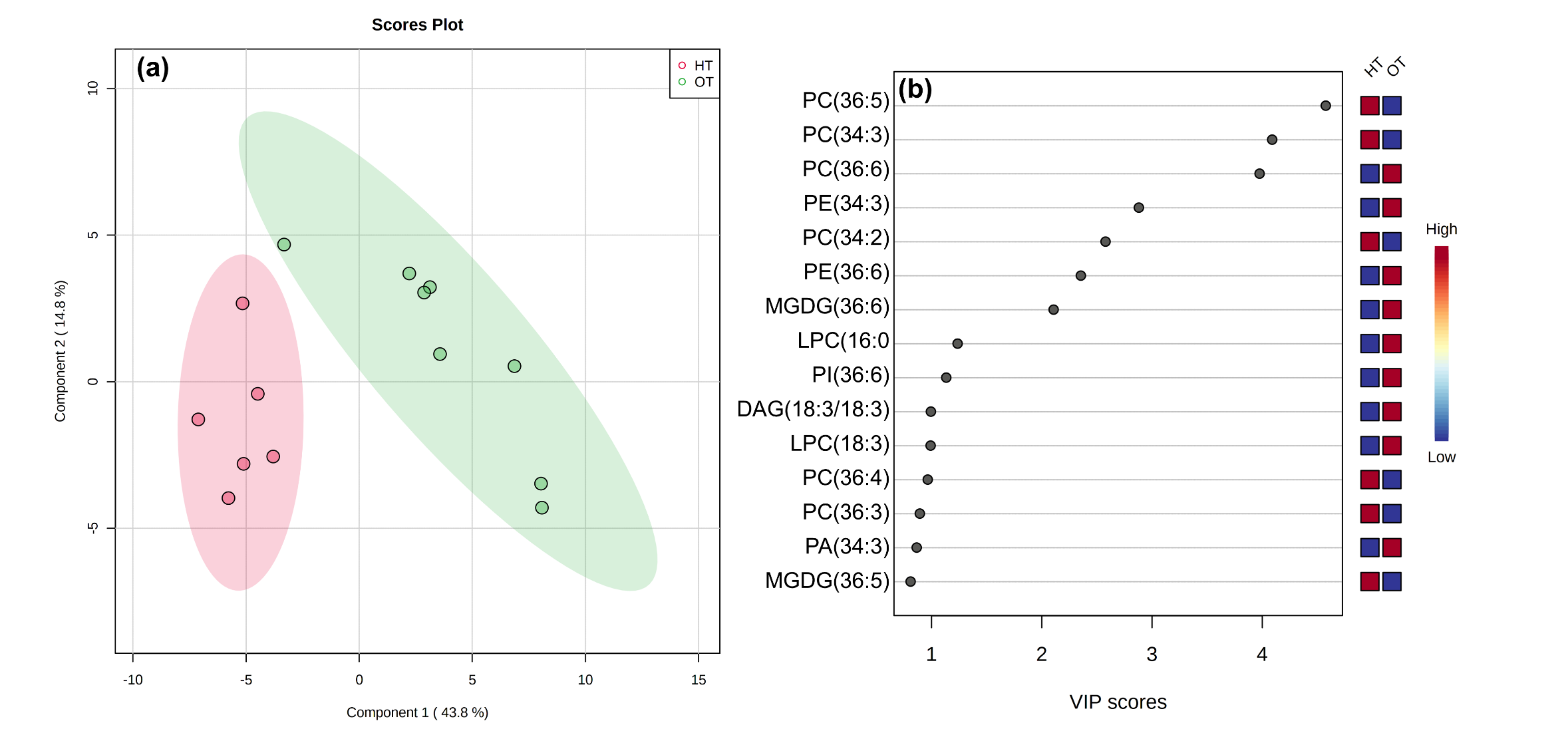
**

**Figure S5.** Partial least squares-discriminant analysis (PLS-DA) scores plot (**a**) visualizes the differentiation of the two treatments [optimum temperature (OT; 25/15°C, day/night) and high day and night temperature (HT; 35/24°C)] PLS-DA variable importance in projection (VIP) scores (**b**) that identified the top 15 lipid species that differentiated OT and HT in the wheat genotype Karl 92. The lipid data presented in these figures were generated by the current authors’ previous research (see Supplementary Table S1 in Narayanan et al., 2018^15^). This data set (Supplementary Table S1, Narayanan et al., 2018^15^) presents the amounts of 89 lipid species measured in pollen samples collected from the heat-susceptible wheat genotype Karl 92, exposed to OT and HT.LPC, lysophosphatidylcholine; MGDG, monogalactosyldiacylglycerol; PA, phosphatidic acid; PC, phosphatidylcholine; PE, phosphatidylethanolamine; PI, phosphatidylinositol.
